# Low-Density Lipoprotein Receptor-Related Protein 1 as a Potential Therapeutic Target in Alzheimer’s Disease

**DOI:** 10.3390/pharmaceutics16070948

**Published:** 2024-07-17

**Authors:** Sabrina Petralla, Maria Panayotova, Elisa Franchina, Gert Fricker, Elena Puris

**Affiliations:** Institute of Pharmacy and Molecular Biotechnology, Ruprecht-Karls-University, Im Neuenheimer Feld 329, 69120 Heidelberg, Germany; s.petralla@uni-heidelberg.de (S.P.); maria.panayotova@uni-heidelberg.de (M.P.); elisa.franchina@studenti.unimi.it (E.F.); gert.fricker@uni-hd.de (G.F.)

**Keywords:** LRP1, Aβ, Alzheimer’s disease, blood–brain barrier

## Abstract

Alzheimer’s disease (AD) is a progressive neurodegenerative disease impacting the lives of millions of people worldwide. The formation of amyloid β (Aβ) plagues in the brain is the main pathological hallmark of AD. The Aβ deposits are formed due to the imbalance between the production and Aβ clearance in the brain and across the blood–brain barrier (BBB). In this respect, low-density lipoprotein receptor-related protein 1 (LRP1) plays a significant role by mediating both brain Aβ production and clearance. Due to its important role in AD pathogenesis, LRP1 is considered an attractive drug target for AD therapies. In the present review, we summarize the current knowledge about the role of LRP1 in AD pathogenesis as well as recent findings on changes in LRP1 expression and function in AD. Finally, we discuss the advances in utilizing LRP1 as a drug target for AD treatments as well as future perspectives on LRP1 research.

## 1. Introduction

Alzheimer’s disease (AD) is an incurable neurodegenerative disease with an increasing prevalence worldwide. The global number of affected individuals is estimated to reach 152.8 million by 2050 [1]. It is the most common type of dementia, accounting for 60–80% of dementia cases [2]. AD cases can be divided into familial Alzheimer’s disease (FAD), caused by a hereditary genetic mutation, and the more common sporadic form of AD (SAD) that does not have a known cause [3,4]. Depending on the age of onset of symptoms being before or after the age of 65 years, AD can be further classified into early-onset (EOAD) and late-onset AD (LOAD) [4,5].

AD is characterized by the presence of two major pathophysiological hallmarks—formation of extracellular amyloid β (Aβ) plaques derived from the amyloid precursor protein (APP) [6,7] and intracellular neurofibrillary tangles (NFTs) consisting of abnormally hyperphosphorylated τ-protein filaments [8,9,10]. As a consequence to these distinct pathological hallmarks, two theories have been developed, describing the sequences of events leading to AD. The first one, the amyloid cascade hypothesis, postulates Aβ as the primary cause of AD, with all other pathological changes, including the NFTs and memory loss, resulting from it [11]. Over 30 years, this theory has been dominant in the AD research field, guiding the focus of drug development programs on targeting either Aβ production or clearance. However, the long-term obstacles in developing effective Aβ-targeting therapies have raised criticism of the Aβ theory [12], leading to re-evaluation of the Aβ deterministic cause–effect model of AD.

The better correlation of NFT pathology with clinical dementia scores compared to Aβ pathology [13] has led to the establishment of the τ theory. This model places the hyperphosphorylation of the microtubule-associated protein τ with subsequent impairment of its ability to bind the neuronal microtubules and induction of self-assembly to NFTs into the center of AD pathology [14]. However, despite the several ongoing studies of τ-targeting therapies, none of them have been approved to be used in clinics [15]. In addition, further factors and pathways have been associated with AD, such as loss of cholinergic neurons [16], oxidative stress [17] and neuroinflammation [18]. Therefore, the current paradigm of AD has changed to being multifactorial and heterogeneous rather than a deterministic model of the disease [19].

The recently approved monoclonal antibodies targeting Aβ clearance have initiated a new era in AD research, confirming the central role of Aβ pathology in AD pathogenesis. However, only a limited population of patients with early stage AD can benefit from these immunotherapies [20]. In addition, the safety profile of the approved monoclonal antibodies should be improved [20]. Therefore, novel, efficient and safe therapeutic strategies to target Aβ clearance are required. In this review, we provide an overview of the current knowledge about the role of the low-density lipoprotein receptor-related protein 1 (LRP1) in AD, a brain endothelial receptor playing a major role in Aβ clearance across the blood–brain barrier (BBB). In addition, we summarize recent findings on LRP1 expression and function in AD and discuss the targeting of LRP1 as a potential modulator of AD pathology.

## 2. Aβ Clearance across the BBB

### 2.1. The Aβ Hypothesis and Aβ Clearance

Extensive research into Aβ in the last three decades, including genetic studies of mutations in the *APP* gene, has provided supportive evidence to the Aβ hypothesis. During non-amyloidogenic processing, APP is cleaved by α- and γ-secretases [21,22]. The cleavage site of the α-secretase is positioned at the 16th amino acid within the Aβ transmembrane domain of APP, which prevents the production of an Aβ monomer [23,24]. If APP is alternatively cleaved by β- instead of α-secretase, a monomeric Aβ peptide is generated, which can further aggregate into plaques [21]. The major Aβ forms are the 40- and 42-residue peptides (Aβ_1–40_ and Aβ_1–42_, respectively). Mutations in and around the Aβ region of *APP* lead to FAD [25,26,27], while mutations in the same *APP* gene that decrease Aβ production are protective against AD [28]. Moreover, mutations in the genes encoding the active site of γ-secretase result in an increased production of Aβ_1–42_, an Aβ subspecies with the tendency to self-aggregate faster [29,30]. 

The concentration of Aβ in the brain depends on the balance between production and clearance of Aβ from the brain and within the brain. The Aβ brain clearance occurs in several ways, including efflux across the BBB to the blood, enzyme-mediated degradation, brain interstitial fluid (ISF) bulk-flow clearance and cerebrospinal fluid (CSF) absorption clearance [31]. Aβ is not only found in AD patients, but also in the CSF and blood of healthy individuals [32]. However, the balance between production and elimination of Aβ protects the healthy brain from Aβ accumulation. Indeed, it has been reported that Aβ in healthy subjects is generated and eliminated from the central nervous system (CNS) at a rate of 7.6% per hour and 8.3% per hour, respectively [32]. On the contrary, in AD patients, Aβ clearance was found to be decreased in the CNS, even when production did not differ from that in healthy controls [33]. Lastly, one of the major genetic predictors of LOAD, the apolipoprotein E (*APOE*)*4* polymorphism, has been linked to decreased Aβ clearance, while the *APOE2* polymorphism has been shown to be protective against AD leading to elevated Aβ clearance [34,35,36,37]. These findings suggest that clearance may play the pivotal role in Aβ accumulation and indicate Aβ clearance as a promising therapeutic strategy for anti-AD drugs. Since there are multiple mechanisms, as well as multiple organs involved in the elimination of Aβ, including the brain and the periphery, this provides an opportunity for a wide variety of therapeutic approaches. 

### 2.2. BBB and the Neurovascular Unit in AD

The BBB, separating the brain from the systemic circulation, is a highly protective barrier formed by the endothelial cells of the brain vasculature [38,39]. The BBB itself acts as a diffusion barrier, maintaining the brain milieu homeostasis. The tight junctions between endothelial cells make the BBB restrictive to most molecules and compounds, with the exception of oxygen, carbon dioxide and some small lipophilic molecules [39,40]. Additionally, multiple ion channels, membrane solute carriers (SLCs) and receptors, expressed at the BBB allow for the ion exchange and the selective transport of nutrients and metabolites across the BBB [39,41]. Moreover, ATP-binding cassette (ABC) transporters, which are highly expressed at the brain endothelial cells, play an important role in maintaining brain homeostasis by restricting the entry of xenobiotics and eliminating potentially toxic metabolites [42].

The endothelial cells of the brain capillaries are further encased by astrocytic end-feet and pericytes [38,43,44]. These different adjoining cell types interact with each other and with neighboring neurons, forming a unit that controls the normal functioning of the CNS, called the neurovascular unit (NVU) [43,45]. Astrocytes and astrocytic end-feet play a role in the neurotransmitter, ion and water homeostasis of the brain, the maintenance of the BBB and the modulation of synaptic transmission [46]. Pericytes have been found to be involved in multiple processes, such as BBB integrity, cerebral blood flow, angiogenesis and clearance of toxins [47,48]. 

In AD, increasing evidence points towards dysfunction of several aspects of the NVU and the BBB. Thus, accumulation of Aβ on the walls of cerebral blood vessels, known as cerebral amyloid angiopathy (CAA), commonly occurs in AD patients [49,50,51]. CAA can cause microbleeds in the brain, which were found to be increased in patients with early-stage dementia, AD patients and APOE4 carriers [52,53,54,55,56] as well as various mouse models of AD [57,58,59]. Moreover, degeneration or dysfunction of other components of the NVU, such as pericytes and astrocytes, as well as the endothelial cells of the BBB themselves, have been reported in AD patients and mouse models of the disease [38,59]. Further signs of BBB damage found in AD patients include reduced cerebral blood flow, increased permeability, leakage of blood-derived proteins and infiltration of peripheral immune cells, such as macrophages and neutrophils, into the brain of AD patients [38]. In addition, studies reported altered expressions of brain endothelial transporters and receptors, which were shown to be involved in Aβ trafficking across the BBB, such as LRP1 and the ABCB1 transporter (also known as P-glycoprotein), which playing a role in Aβ clearance from the brain, and the receptor for advanced glycation end products (RAGE), which controls the Aβ influx from systemic circulation [60,61,62]. All these vascular alterations can affect the clearance of Aβ from the brain.

### 2.3. Clearance of Aβ across the BBB 

The exact mechanism of Aβ clearance across the BBB is still not well understood. Studies have demonstrated that LRP1, expressed at the abluminal, brain-facing side of the brain capillary endothelial cells, plays a major role in this process [63]. LRP1 rapidly removes soluble Aβ from the ISF by both endocytosis and lysosomal degradation inside the brain capillary endothelial cells as well as by transcytosis across the endothelial cells [63,64]. The role of LRP1 in Aβ clearance across the BBB is discussed in more detail in Section 4.5. In addition, ABCB1, an efflux transporter expressed at the luminal side of the brain capillary endothelial cells, was shown to play a critical role in Aβ trafficking across the BBB [65,66,67,68]. Interestingly, Strock et al. demonstrated that both LRP1 and ABCB1 cooperate and cannot mediate removal of Aβ across the BBB on their own [69]. According to the authors of that study, after endocytosis of Aβ at the abluminal side by LRP1, ABCB1 is recruited to the sorting endosome, which may represent a vesicle transferring Aβ to the luminal side [69].

In addition to ABCB1, other ABC and SLC transporters have demonstrated potential roles in Aβ clearance from the brain [70,71]. Moreover, other Aβ clearance mechanisms across the BBB have been proposed, including LRP2-mediated removal of Aβ bound to apolipoprotein J (APOJ) [72]. However, the contribution of each of these mechanisms to the total Aβ clearance from the brain and their role in AD pathology remain unknown. 

## 3. Peripheral Clearance of Aβ 

To avoid potential side effects related to brain Aβ-targeting therapies, an alternative approach of the peripheral Aβ removal has been proposed [73]. This strategy is based on the idea that the concentration of Aβ in the brain and periphery exists in a state of equilibrium. Consequently, increasing the clearance of Aβ from the blood and peripheral organs could act as a “sink”, causing efflux of Aβ from the brain into the periphery and thus reducing the brain load. This concept, called the “peripheral sink hypothesis” [74,75], has been a controversial topic in AD research for the past two decades, with conflicting results from different research groups. On the one hand, some studies suggest that reduction in Aβ in the periphery does not affect the brain Aβ load [76,77,78] or even if it decreases brain Aβ levels, it does not eliminate AD symptoms [79]. In contrast, several studies have supported the peripheral sink hypothesis, including biodistribution studies using ^125^I-labelled Aβ in wild type (WT) rats [80] and in the APPswe/PSEN1dE9 mouse AD model [81]. Moreover, peripheral administration of an anti-Aβ antibody in the PDAPP mouse model of the disease [74] and of an Aβ sequestering agent in a PS/APP mouse model [82] were found to lower the Aβ brain load without crossing the BBB, which suggests a mechanism of action through an increase in the efflux of Aβ from the brain. Furthermore, the removal of Aβ from the blood via whole blood exchange in the APPSwe (Tg2576) mouse model of AD [83] as well as plasma exchange and peritoneal dialysis in the APPswe/PSEN1dE9 mouse model [84,85] has been reported to lower brain Aβ concentrations. In these studies, the whole blood exchange also resulted in improved memory [83], while the peritoneal dialysis reduced neuroinflammation, neurodegeneration and cognitive decline in mice [85]. Additionally, hemodialysis has been associated with lower Aβ brain load in humans [86], while plasma exchange has been shown to improve cognitive deficits in patients with AD [87,88]. Finally, transfer of peripheral Aβ from APPswe/PSEN1dE9 mice into WT mice via parabiosis [89] or via the transplantation of Aβ-expressing bone marrow cells [90] was found to induce neurodegeneration and formation of amyloid plaques in the WT mice. The opposite effect was also observed, with the reduction in peripherally produced Aβ by replacing the bone marrow cells of APPswe/PSEN1dE9 mice with those from WT mice resulting in lower Aβ brain burden [90]. 

## 4. The Role of LRP1 in AD

### 4.1. LRP1 Structure, Expression and Function

LRP1 is a type 1 transmembrane protein that is a part of the low-density lipoprotein receptor (LDLR) family. LRP1 is synthesized as a 600 kDa precursor protein that is proteolytically cleaved by Furin in the Golgi complex in an extracellular α-chain of 515 kDa and a β-chain of 85 kDa [91,92,93]. Its structure has been previously described in detail [94,95,96]. However, it is essential to mention the presence of four ligand-binding domains (I–IV) in the extracellular domain, of which the domains II and IV are the major binding regions [97,98]. Moreover, the cytoplasmic tails of LRP1 include two NPXY motifs and one YXXL motif, which play an important role in the endocytosis mechanism [99,100]. Finally, LRP1 is also present in a soluble form (sLRP1) due to cleavage by α- and β-secretase [94].

LRP1 was first discovered during screening of human hepatic cDNA libraries and found to be highly expressed in the liver [101]. The same study observed high levels of *Lrp1* mRNA expression in the lung, brain, intestine and muscle tissues of mice, as well as detectable levels in the heart, kidney, spleen, bone and thymus [101]. Following studies have confirmed that LRP1 is abundantly expressed in multiple organs in humans, including the liver, brain, lungs, intestines and the vascular system [102], as well as circulating in plasma in the form of sLRP1 [103], and that it plays a key role in the transport and clearance of substances, as well as signaling and regulation of biological processes (extensively reviewed in Lillis et al. [95]). In the brain and at the NVU, LRP1 is expressed in multiple cell types. Bu et al. [104] observed mRNA expression of the *Lrp1* gene in neurons of the granular cell layer of the cerebellum and large Purkinje cells, the dentate granule cell and pyramidal cell layers in the hippocampus and in the cortex of the rat brain using in situ hybridization. Further immunostaining studies in human postmortem samples have revealed strong LRP1 protein expression in the neuronal cell bodies and proximal processes of the granule neurons of the dentate gyrus and pyramidal neurons of the hippocampus, temporal neocortex, cerebral cortex and thalamus and weak LRP1 protein expression in astrocytic foot processes [105,106]. Interestingly, Rebeck et al. [105] also compared healthy controls and AD patients and found strong LRP1 staining in reactive astrocytes and amyloid plaques in AD patients additional to that of neurons. Moreover, at the NVU, LRP1 protein expression has been detected using immunostaining in the pericytes [106,107,108] and in the endothelium [108,109,110], mainly at the abluminal side of the BBB [109,111]. 

Given its expression in many tissues, it is not surprising that the functions of LRP1 are also numerous. Indeed, LRP1 is involved in various physiological processes, such as lipid and glucose metabolism, protease degradation and energy homeostasis, as well as in several pathogenic conditions, including inflammation, cancer, atherosclerosis and AD [95]. LRP1 can bind more than 40 different ligands, mainly through its extracellular domains II and IV, including Aβ, APOE and α2- macroglobulin, mediating and regulating their transport through receptor-mediated transcytosis (RMT) [112]. The RMT process includes binding of a ligand to LRP1 and consequently inducing the endocytosis pathway in which the ligand–receptor complex is internalized into an intracellular vesicle. Once internalized, the vesicle containing the ligand–receptor complex fuses with the plasma membrane in the exocytosis process. The ligand, dissociated from the receptor, may be degraded into lysosomes, recycled via the endosome or released on the abluminal surface, while the receptor is recycled back to the cell surface [96]. The NPXY and YXXL motifs present in the cytoplasmic tails of LRP1 are highly involved in this process as dominant signals for LRP1 endocytosis [99]. In addition to its crucial role in RMT across the BBB, LRP1 has a wide variety of other functions—as a regulatory, scavenger and scaffold receptor. It can regulate transcription and gene expression after translocation of its intracellular domain in the nucleus or it can act as a scaffold for intracellular adaptor proteins [96,113]. Significantly, in the context of AD, LRP1 is believed to play a role in both production and clearance of Aβ, as discussed in the following sections.

### 4.2. Expression of LRP1 in AD

Several studies have investigated the association of AD pathology with changes in the expression of LRP1 in the brain. Evidence on LRP1 expressional alterations has been reported in AD patients (Table 1) as well as in rodent models of AD (Table 2). Jeynes et al. [114] reported increased levels of LRP1 in the cerebral capillaries of AD patients compared to non-demented controls (NDCs). In another study, increased hippocampal LRP1 levels in the microvasculature, but reduced LRP1 levels in neurons in AD patients were reported [110]. In addition, elevated LRP1 levels were observed in brain regions strongly implicated in AD, such as the hippocampus and the frontal cortex of AD patients as compared to NDC [110,115,116]. The increased expression of brain LRP1 observed in these studies can be a compensatory mechanism to promote Aβ clearance [117]. On the other hand, no significant differences in the brain cortical, hippocampal, astrocytic and cerebral capillary LRP1 levels between AD patients and NDC were reported in other studies, although a significant positive correlation between LRP1 capillary expression and plaque burden was observed [118,119,120,121,122]. In contrast, decreased levels of LRP1 were observed in the cerebellum, occipital and midfrontal cortex of AD patients [115,123,124]. Interestingly, a decrease in sLRP1 levels in plasma was reported in AD patients compared to NDC with no correlation between plasmatic Aβ and sLRP1 found in AD patients [125]. All in all, these studies indicate that LRP1 expressional changes can be brain region- and cell-specific as well as dependent on the stage of the disease. 

**Table 1:** Low-density lipoprotein receptor-related protein 1 (LRP1) changes in Alzheimer’s disease (AD) patients as compared to non-demented controls (NDC).

Subjects (Age in Year Range; Number of Subjects per Sex)	AD Patients	Investigated Samples	Findings AD vs. NDC	Methods	References
NDC (41–75 y; *n* = 5/M, *n* = 5/F) AD (74–88 y; *n* = 6/M, *n* = 4/F)	Braak stage 1–6; CERAD index Moderate-High	Capillaries of superior temporal and calcarine occipital cortex	Increase in the number of LRP1-positive capillaries; negative correlations between LRP1-positive capillaries and NFTs and Aβ plaque burden	IHC	[114]
NDC (68 y, *n* = 15) AD (79 y, *n* = 15)	Braak stage 4–6; CERAD index High	Capillaries of temporal cortex	No difference in LRP1 capillary expression; positive correlations between Aβ_1–42_ plaque burden and positive LRP1 capillary expression	IHC	[118]
Controls (*n* = 19) AD (*n* = 23)	Braak stage 4–6; CERAD index High; none/mild CAA (n = 27) moderate/ severe CAA (n = 15)	Frontal and occipital cortex; occipital and frontal leptomeningeal vessels	No difference in LRP1 expression in relation to CAA or between AD and controls	Dot blot	[123]
NDC (60–88 y; *n* = 10/M, *n* = 10/F) AD (78–89 y; *n* = 5/M, *n* = 5/F)	Braak stage 3–5; CERAD 1–3	Perivascular astrocytes (basal ganglia)	No difference in LRP1 expression in perivascular astrocytes	IHC	[120]
NDC (60–68 y; *n* = 264/M, *n* = 461/F) AD (68–77 y; *n* = 34/M, *n* = 36/F)	MMSE 18	Plasma	Reduction in sLRP1 levels	ELISA	[125]
NDC (75–79 y; *n* = 9) AD (77–82 y; *n* = 8)	Braak stage High	Frontal cortex	Increased LRP1 levels in AD	Western blot	[116]
Controls (*n* = 20) AD (*n* = 38) probable-AD (*n* = 3)	CDR 4–5	Frontal cortex	No difference in LRP1 levels	Western blot	[119]
Controls (40–64 y, *n* = 11; 65–74 y, *n* = 11; 75–84 y, *n* = 11; ≥ 85 y, *n* = 6) AD (65–74 y, *n* = 9; 75–84 y, *n* = 22; ≥85 y, *n* = 8).	LOAD	Midfrontal cortex	Reduction in LRP1 levels in older age and in AD	Western blot	[124]
NDC (82–89 y; *n* = 6/M, *n* = 3/F) AD (85–89 y; *n* = 5/M, *n* = 4/F)	Braak stage 4–6	Hippocampus	No difference in LRP1 levels	Western blot, IP	[121]
NDC (70–78 y; *n* = 3/M, *n* = 3/F) AD (79–86 y; *n* = 3/M, *n* = 3/F)	Braak stage 5–6	Hippocampus	Increased LRP1 levels in the microvasculature, reduced LRP1 levels in neurons	IHC, Western blot	[110]
NDC (81–88 y; *n* = 17/M, *n* = 21/F) AD (84–91 y; *n* = 21/M, *n* = 22/F)	Braak stage 4–6	Cerebellum, BA39, Hippocampus	Reduction in LRP1 levels in cerebellum, increased LRP1 levels in BA39 and hippocampus	LC-MS/MS	[115]
NDC (66–82; *n* = 16) AD (68–88: *n* = 17)	Disease duration 5–10 y	Temporal cortex	No difference in LRP1 levels	ELISA	[122]

Aβ—amyloid β; BA39—parietal lobe; CAA—cerebral amyloid angiopathy; CDR—clinical dementia rating; CERAD—The Consortium to Establish a Registry for Alzheimer’s Disease; ELISA—enzyme-linked immunosorbent assay; F—female; IHC—immunohistochemistry; IP—immunoprecipitation; LC-MS/MS—liquid chromatography coupled to tandem mass spectrometry; LOAD—late-onset Alzheimer’s disease; M—male; MMSE—Mini-Mental State Examination; NDC—non-demented control; NFTs—neurofibrillary tangles; OC—occipital calcarine; sLRP1—soluble LRP1; SO—striate occipital; ST—superior temporal; y—years.

Interestingly, the majority of studies in mouse and rat models of AD revealed a significant reduction of LRP1 expression levels in the brain, as well as in the intestine and liver [126,127,128,129,130,131,132,133]. In contrast, increased LRP1 levels in the choroid plexus were found in 3xTg-AD mice [134]. Interestingly, brain microvascular and parenchymal LRP1 protein levels in APOE transgenic mice were not different between APOE genotypes [135]. As the studies of LRP1 expression in AD models were performed at the stage when AD-related pathology has been developed, it is unclear if the LRP1 reduction is a result of these pathological changes or if it triggers them. In addition, no studies comparing sex-related changes in LRP1 expression have been reported. Therefore, future studies should address these aspects and reveal the role of LRP1 in the manifestation and development of AD pathology with consideration of sex. 

**Table 2:** Changes in LRP1 protein levels in animal models used in AD research.

Model (Number of Animals; Age; Sex)	Investigated Samples	Findings	Methods	References
*Mice*				
APP/PS1 (*n* = 10; 3–15 m; M)	Cerebral cortex and hippocampus	Progressive reduction in LRP1 levels with ageing	Western blot	[128]
APP/PS1 (*n* = 3; 8–9 m; F)	Intestine, Liver	Reduction in LRP1 expression in intestine, no difference in the liver	IHC	[126]
APP/PS1 (*n* = 3; 3 m; M)	Hippocampus	Reduction in LRP1 levels	Western blot	[127]
APP/PS1 (*n* = 10; 12 m; M)	Brain	Reduction in LRP1 expression	Western blot	[129]
SAMP8 (*n* = 8; 11 m)	Cortex and hippocampus	Reduction in LRP1 expression	IF, Western blot	[133]
3xTg-AD (*n* = 3; 24 m)	Hippocampus	Reduction in LRP1 levels	IHC, Western blot	[130]
3xTg-AD (*n* = 8; 16 m)	Choroid plexus	Increase in LRP1 expression	IF	[134]
*Rat*				
ALAD (*n* = 7; 8–10 wk; M)	Brain	Reduction in LRP1 levels	ELISA	[131]
SD + Aβ_1–42_ (*n* = 8; 8 wk; M)	Brain	Reduction in LRP1 expression	Western blot, IHC	[132]

APP—amyloid precursor protein; ALAD—AlCl3-induced Alzheimer’s disease; CEC—cerebromicrovascular endothelial cells; IF—immunofluorescence; m—months old; PS1—presenilin 1; SAMP8—senescence-accelerated mouse prone-8; SD—Sprague–Dawley; SDZ—streptozotocin; wk—weeks old; Tg—transgenic.

Multiple studies have explored the impact of Aβ on LRP1 expression in brain endothelial cells in vitro (Table 3). LRP1 levels were decreased by both Aβ_1–40_ and Aβ_1–42_ treatment (ranging from 50 nM to 5 μM) of human brain endothelial cells hCMEC/D3 as well as of primary porcine brain capillary endothelial cells (pBCECs) [136,137,138]. In primary human brain microvascular endothelial cells (HBMECs), a significant increase of sLRP1 in the extracellular media was observed after Aβ_1–42_ treatment (2 μM) [139,140]. LRP1 is a ubiquitously expressed endocytic receptor on the cell surface, but has a soluble and circulating form, sLRP1, which is generated from LRP1. sLRP1 has been detected in the circulation and in other body fluids, and several stimuli have been identified that lead to the processing of LRP1 in its soluble form. In these cases, it appears that exposure to Aβ causes a reduction in LRP1 on the cell surface of the BBB, leading to an increase in its soluble form, sLRP1 [135]. This would suggest a negative correlation between LRP1 expression and the higher levels of Aβ found in the brain of AD patients. Therefore, the accumulation of Aβ in the brain could be a consequence of reduced LRP1 expression. 

**Table 3:** In vitro studies on LRP1 protein expression changes in BBB models.

Cell Line	Treatment	Findings	Methods	References (DOI)
HBMECs	Aβ_1–42_ 2 μM 48 h	Increase in sLRP1 levels	ELISA	[139]
HBMECs	Aβ_1–42_ 2 μM 48 h	Increase in sLRP1 levels	ELISA	[140]
HBMECs	Aβ_1–42_ ≥2 μM 48 h	Increase in sLRP1 levels but no significant difference in LRP1 expression	ELISA	[135]
HBMECs	Aβ_1–42_ 10 μM 48 h	No difference in LRP1 expression	ELISA, Western blot	[141]
hCMEC/D3	Aβ_1–42_ 1 μM, 5 μM 2 h, 6 h	Reduction in LRP1 expression	Western blot	[137]
hCMEC/D3	Aβ_1–40_, Aβ_1–42_ 100 nM, 50 nM 24 h	Reduction in LRP1 expression	Western blot	[138]
pBCECs	Aβ_1–40_, Aβ_1–42_ 240 nM 6 h	Reduction in LRP1 expression	Western blot	[136]

HBMECs—primary human brain microvascular endothelial cells; hCMEC/D3—human brain endothelial cells; pBCECs—primary porcine brain capillary endothelial cells.

### 4.3. Genetic Studies on LRP1 in AD

Several potential AD susceptibility genes have been reported in genome-wide association studies (GWASs) [142,143,144,145]. Among them, great interest has been reported for *Lrp1*, whose single-nucleotide polymorphisms (SNPs) have been extensively investigated and associated with AD [92]. However, genetic studies on the association between *Lrp1* polymorphisms and AD risk are controversial. C766T polymorphism in exon 3 (rs1799986) of the *Lrp1* gene was the first reported polymorphism and is the most studied to date [146,147]. This is a silent mutation that does not affect the amino acid sequence of the receptor [146]. Initially it was reported as an underrepresented polymorphism in LOAD [146,147]. However, recently, several meta-analysis studies showed that this polymorphism does not affect AD susceptibility individually [92,148,149,150] despite being significantly associated with CAA [151]. Only the interaction of *Lrp1* C766T polymorphism with other genes involved in AD pathogenesis, such as *APOE* and *MAPT* (microtubule associated protein tau), has been reported to be a risk factor for AD predisposition [152,153,154,155,156]. Additionally, Shi et al. reported a significant association between *Lrp1* haplotypes and mild cognitive impairment (MCI), which represents the early stages of AD [157].

### 4.4. Role of LRP1 in Production of Aβ

LRP1 has been found to affect the production of Aβ. It can mediate the internalization of APP [158,159] and its blocking in APP-transfected H4 human neuroglioma cells leading to higher levels of cell surface APP and lowered Aβ production [160]. Moreover, overexpression of a LRP1 minireceptor resulted in lower cell surface APP levels in Chinese hamster ovary (CHO) cells [161]. In the brain of PDAPP transgenic mice, overexpression of LRP1 minireceptor led to an age-dependent increase in soluble brain Aβ and memory decline [162]. Furthermore, Lakshmana et al. [163] reported that C-terminal residues of LRP1 could promote the processing of APP to Aβ in APP-transfected human embryonal kidney (HEK) 293T cells. Yoon et al. observed an increase in Aβ generation in CHO co-transfected with APP and LRP1 C-terminal residues [164]. In contrast, Lleó et al. found that overexpression of LRP1′s C-terminal transmembrane domain in cells transfected with APP reduced Aβ production by competing with APP over γ-secretase cleavage [165]. Finally, a recent in vivo study confirmed that inactivation of LRP1 in mice resulted in reductions in both the clearance and production of Aβ, with the effect on its generation being predominant and thus leading to an overall decrease in Aβ [166]. In summary, multiple studies have established the involvement of LRP1 in the production of Aβ. 

### 4.5. LRP1-Mediated Clearance of Aβ 

**Figure 1:**
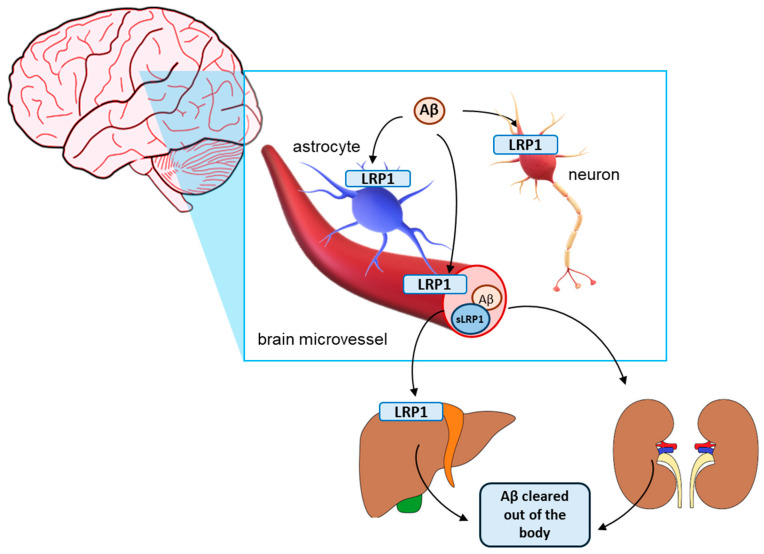
Roles of the low-density lipoprotein receptor-related protein 1 (LRP1) in the clearance of amyloid β (Aβ). In the brain, LRP1 is implicated in the uptake for subsequent degradation of Aβ in neurons, astrocytes and vascular smooth muscle cells. Moreover, LRP1 plays a key role in the efflux of Aβ from the brain into the blood through the brain microvascular endothelial cells. Aβ then enters the systemic circulation where it can further be cleared by soluble LRP1 (sLRP1), freely circulating in the plasma. Aβ is further transported into peripheral clearance organs, such as the liver, with the help of LRP1 and is further cleared from the body.

The second main aspect of the role of LRP1 in AD is the clearance of Aβ. LRP1 is involved in both the efflux of Aβ from the brain and periphery and in its endocytosis and degradation in neurons and astrocytes (Figure 1). In fact, LRP1 is the main receptor involved in the brain Aβ clearance across the BBB [167]. LRP1-mediated clearance of Aβ was found to be a very rapid process in the murine CNS and to even occur six times faster than Aβ clearance via the ISF flow for the Aβ_1–40_ species [72]. LRP1 binds Aβ directly or indirectly by interacting with other factors, thus beginning its clearance across the BBB. APOE and clusterin have been reported as major players in this process. However, recently, GWASs have identified phosphatidylinositol-binding clathrin assembly protein (PICALM) as a new and prominent factor involved in the process of endocytosis of the Aβ-LRP1 complex [69,111]. Reduced PICALM expression levels have been reported in cerebral microvessels of AD patients as well as in Picalm+/− mouse model, confirming a central role of PICALM in the exacerbation of Aβ pathology by reducing cerebral Aβ clearance [69,111]. Inhibition of LRP1 has been shown to drastically decrease the clearance of ^125^I-labelled Aβ from the brain of WT mice [168]. Chronic antisense inhibition of LRP1 via intracerebroventricular infusion not only reduced Aβ efflux from the brain but it also led to memory impairment in WT mice [169]. Furthermore, deletion of LRP1 in brain endothelial cells in WT and 5xFAD mice was found to lead to reduced Aβ efflux from the brain [63] and its conditional knockout in vascular smooth muscle cells [64], neurons [170] and astrocytes [171] of APPswe/PSEN1dE9 mice decreased Aβ uptake and degradation in these cell types and increased Aβ brain accumulation. Moreover, LRP1 deletion paired with lysosomal enzyme inhibition impaired Aβ degradation in cultured human brain vascular smooth muscle cells (HBVSMCs) [64]. This effect was not observed during inhibition of lysosomal enzymes only, showing LRP1’s role in local Aβ degradation [64]. In addition, downregulation of LRP1 in cultured mouse primary astrocytes not only affected Aβ clearance but also reduced the level of Aβ-degrading enzymes, signifying that LRP1 may also affect extracellular Aβ metabolism [171].

Additionally, LRP1 possibly plays a role in Aβ clearance at the CSF, as Fujiyoshi et al. found that administration of the LRP1 antagonist receptor-associated protein (RAP), LRP1 ligand α2-macroglobulin and anti-LRP1 antibodies decreased the efflux of intracerebroventricularly administered ^125^I-labelled Aβ from the CSF of WT rats [172].

The fact that sLRP1circulates in the blood and binds the substantial part of plasma Aβ in healthy individuals makes it a vital player in the peripheral clearance of Aβ. The levels of sLRP1 in the plasma were found to be reduced in AD patients, as well as in patients with MCI. Moreover, much of the remaining sLRP1 was found to be oxidized, resulting in a lower binding affinity for Aβ [173,174]. One study also found that carriers of the APOE4 variant, one of the main LOAD risk factors, had reduced levels of sLRP1 [175]. Furthermore, a decrease in sLRP1-bound Aβ in the plasma was even proposed as a biomarker for MCI patients that later develop AD [174]. Finally, peripheral administration of recombinant LRP1 cluster IV, one of LRP1’s major binding domains, in mice heterogenous for the Swedish mutation of APP (APP^+/sw^) was found to reduce the Aβ brain load without crossing the BBB [173].

The precise mechanism of peripheral clearance of Aβ is not fully understood. However, it has been shown that most of the Aβ in the periphery is cleared by the liver, followed by the kidney [176,177,178]. It is believed that LRP1 plays a major role in the hepatic clearance of Aβ. Suppression of the hepatic LRP1 expression in RAP-deficient mice or using small interfering RNA (siRNA) treatment in WT mice, as well as LRP1 inhibition with RAP in WT rats, resulted in a significant reduction of the transport of ^125^I-labelled Aβ from the blood into the liver [179]. Moreover, knockdown of hepatic LRP1 expression in APPswe/PSEN1dE9 mice was shown to not only lead to higher concentration of Aβ and amyloid plaques in the brain but it also worsened the behavioral decline and increased other AD-related pathological features, including neurodegeneration, neuroinflammation and τ hyperphosphorylation [180]. On the other hand, increasing the LRP1 expression in the hepatocytes of APPswe/PSEN1dE9 mice had an opposite effect—it decreased the amount of Aβ and hyperphosphorylated τ in the brain, reduced neurodegeneration and neuroinflammation and improved cognitive deficits [180,181]. In view of the newly emerging research implying both the importance of peripheral Aβ clearance in AD pathology and the role of LRP1 in that process, targeting LRP1 in the periphery can be a promising therapeutic approach to treat AD. 

### 4.6. Role of LRP1 in τ Pathology

Finally, recent studies have associated LRP1 with τ endocytosis and propagation. LRP1 knockdown in H4 neuroglioma cells and neurons derived from induced pluripotent stem cells was shown to decrease τ uptake and its downregulation in WT mice reduced the spread of τ [182]. LRP1 was also shown to mediate the internalization of ^125^I-labelled τ and to promote τ propagation in cell culture [183]. Additionally, a genetic interaction between the intron 9 rs2471738 polymorphism of τ and the exon 3rs1799986 *Lrp1* polymorphism was detected in AD patients and individuals with a T allele in both polymorphisms were found to be 6.2 times more likely to develop AD [154].

## 5. LRP1 as Therapeutic Target

**Figure 2:**
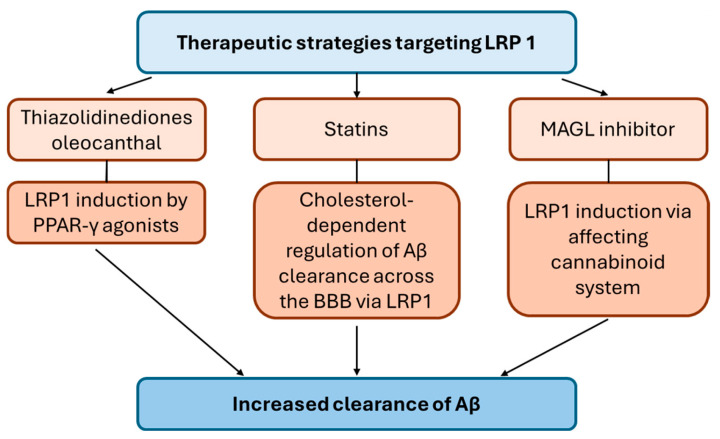
Reported potential therapeutic strategies targeting the low-density lipoprotein receptor-related protein 1 (LRP1) to increase the clearance of amyloid β (Aβ) across the blood–brain barrier (BBB) including a peroxisome proliferator-activated receptor gamma (PPAR-γ) agonists, cholesterol-lowering statins and a monoacylglycerol lipase (MAGL) inhibitor.

The important role of LRP1 in AD pathogenesis makes it an attractive drug target for AD treatment. Thus, several studies investigated whether modulating LRP1 expression at the BBB could facilitate Aβ brain clearance and reduce the accumulation of soluble Aβ in the brain, potentially slowing AD progression (as shown in Figure 2). For instance, rosiglitazone, a peroxisome proliferator-activated receptor gamma (PPAR-γ) agonist, increased LRP1 expression at low concentrations in HBMECs, which resulted in greater LRP1-mediated transport of Aβ in this in vitro BBB model [184]. Based on the results of this study, the authors proposed using low doses of insulin sensitizers thiazolidinediones, such as rosiglitazone and pioglitazone, for AD treatment. Indeed, Seok et al. demonstrated that administration of low doses of pioglitazone in senescence-accelerated mouse prone-8 (SAMP8) mice induced hippocampal LRP1 expression leading to decreased Aβ deposits and Aβ_1–40_ levels as well as reversed learning and memory impairment in mice [133]. Interestingly, in a recent study by Zhao et al., the use of thiazolidinediones was associated with a lower risk of dementia incidence in a mainland Chinese population with type 2 diabetes mellitus [185]. However, the effect of thiazolidinediones on LRP1-mediated Aβ clearance in the brain of these patients has not been studied.

In addition, administration of cholesterol-lowering statins was investigated to facilitate the Aβ clearance via LRP1 expression modulation in AD. Thus, Shinohara et al. revealed dose-dependent increase in LRP1 expression after treatment of HBMECs with fluvastatin [186], which resulted in elevated Aβ uptake. In the same study, administration of fluvastatin in APP transgenic mice (APP23) reduced brain levels of Aβ, which was associated with increased LRP1 expression in the brain microvessels of mice. In another study, 3-week treatment of 3xTg AD mice with simvastatin increased *Lrp1* and *Lrp2* gene expression in isolated brain microvessels [187]. In addition, simvastatin treatment upregulated LRP1 protein levels in primary pBCECs [187]. Interestingly, Zhou et al. observed decreased expression of LRP1 in the isolated brain microvessels of high cholesterol an AD mouse model associated with disturbed Aβ transport [188], providing the evidence of cholesterol-dependent regulation of Aβ clearance across the BBB via LRP1. This study supports the use of cholesterol-lowering statins in AD. However, the results of the clinical trials on the benefits of the statin use in AD are conflicting [189]. Nevertheless, there is evidence that administration of cholesterol-lowering therapies, such as statins in mid-life, can lower the risk of dementia [190]. In addition, it was proposed that statin therapy may be beneficial for specific population of AD patients, such as those homozygous for APOE4 [191]. Although most of the proposed mechanisms associated to the statin effects in AD are related to the cerebrovascular system, statin administration was shown to increase hepatic LRP1 expression [192], which can facilitate peripheral clearance of Aβ. Future studies should focus on examination of the specific mechanism of statin effects in AD, which could help in identifying the populations of patients who can benefit from this treatment.

Other LRP1-targeting compounds have been tested to increase Aβ clearance. For instance, administration of oleocanthal, a phenolic secoiridoid component of extra-virgin olive oil, in TgSwDI mice led to a decrease in Aβ burden in the mouse hippocampus as well as enhanced ^125^I-Aβ_40_ clearance across the BBB, which was associated with upregulated LRP1 and ABCB1 expression in the mouse brain microvessels via PPARγ activation [193]. In addition, Bachmeier et al. presented an interesting approach of targeting LRP1-mediated clearance of Aβ via affecting the cannabinoid system [194]. Thus, the authors demonstrated enhanced brain and plasma expression of LRP1 as well as increased clearance of intracerebrally administered Aβ from the brain to the periphery in WT mice treated with a monoacylglycerol lipase (MAGL) inhibitor, regulating levels of endocannabinoid 2-arachidonoyl-glycerol [194]. 

## 6. Future Perspectives and Conclusions

In the present review, we summarized the most recent information about the role of LRP1 in AD pathogenesis with an emphasis on its contribution to Aβ clearance and use as a potential drug target for AD treatments. From our literature research, although several studies investigated changes in LRP1 protein levels in the brain of human and rodent AD models, numerous shortcomings were found. Almost all studies have explored the whole brain or just some brain regions such as the frontal cortex and hippocampus, while only a few studies have focused on investigating the changes in LRP1 expression in brain microvessels. In addition, no sex-specific alterations in LRP1 expression have been reported in either AD patients or animal models. Moreover, there is a lack of information about the time course of the changes in LRP1 expression in AD, which makes it difficult to elucidate the contribution of these alterations in AD pathogenesis. Therefore, future studies should focus on the analysis of LRP1 expressional and functional alterations of individual brain regions with the emphasis on sex as well as on the elucidation of the impact of these changes on AD manifestation and development.

As previously discussed, several attempts have been made to develop LRP1-targeting therapies for AD. However, none of them are currently in clinical use. One of the reasons is that LRP1 is not the only player in the Aβ clearance. As mentioned before, ABC transporters have been shown to play an important role in this process. However, there are still big gaps in our knowledge about the contribution of all players, their specific roles, and interconnections in the Aβ brain clearance. In addition, due to limited information about the role of LRP1 in AD manifestation and development, it is difficult to evaluate at which stage of AD the patients could benefit from LRP1 modulators. Furthermore, although different approaches have been proposed for modulating LRP1, most of the studies provide only “association-based” evidence of LRP1 modulation. Thus, the mechanisms of the proposed LRP1-modulating therapies should be further investigated so they can be tested in clinical trials. Importantly, although recent studies have revealed potential roles of the liver and kidney in the process of circulating Aβ clearance, there is a lack of evidence of direct involvement of LRP1 in renal and hepatic Aβ transport. Further studies are needed, especially because removal of peripheral Aβ could be a promising therapeutic strategy for AD [176,177,178]. Finally, recent studies have demonstrated a substantial involvement of LRP1 in τ pathology. A critical role of LRP1 in the spread of τ in the brain has been reported, as well as a significant association between *Lrp1* and *τ* polymorphisms and AD risk [182,183]. However, the mechanism of the regulation of τ spreading by LRP1 still remains unclear. So, further investigation is needed for proposing a potential therapeutic intervention for AD that acts on both plaque and tangle pathologies. All in all, although LRP1 is a promising drug target for AD treatment, the process of developing effective therapies targeting LRP1-mediated Aβ clearance would require more thorough investigation of the role of LRP1 in AD.
